# Supplementary material for: Building non-motorized accessible communities for heterogeneous demand: A facility location and network design problem
Source: PLoS One. 2024 Oct 17;19(10):e0312230. doi: 10.1371/journal.pone.0312230 (PMC11486433; doi:10.1371/journal.pone.0312230)
Supplement: Attachment — Submitted filename: Review Report_PONE-D-24-23480_v1.pdf [file pone.0312230.s001.pdf]

Referee Report Regarding the Manuscript:

PONE-D-24-23480

Building Non-Motorized Accessible Communities for Heterogeneous Demand: A Facility Location and Network Design Problem

Submitted to:

PLOS ONE

A. Brief Summary

This manuscript presents an optimization model to enhance non-motorized accessibility in urban environments by identifying optimal locations for service facilities and accessible road segments. The study uses a randomized greedy algorithm to address a complex network design and facility location problem, demonstrating its application through a case study in the Huai Kwang district of Bangkok. This research is timely due to increasing urban mobility issues and aims to cater to diverse user needs, enhancing access to essential services like healthcare and education.

B. Overall Impression

The paper is well-structured and addresses a significant and timely issue in urban planning. The combination of facility location theory and network design to enhance non-motorized accessibility is innovative and has the potential to contribute significantly to the field. However, the manuscript could benefit from a clearer exposition in some sections, more rigorous validation of the results, and deeper integration of the literature to position its contributions more distinctly within the current research landscape.

C. Major Comments

1. **Scientific Merits and Consistency of Arguments**
    - The manuscript effectively frames the problem within the context of social justice and environmental sustainability. However, the linkage between the proposed model and existing theories could be strengthened. For instance, the discussion on non-motorized accessibility measures could be better integrated with the model's

objectives to highlight its innovation (e.g., how the proposed measures improve upon or differ from those like distance-based or gravity-based measures).

- The methodological explanation in Sections 2 and 3 is complex and could benefit from additional diagrams or flowcharts to aid in understanding the model's workflow and algorithmic steps.
- In regards to the optimization model, the assumptions and limitations should be explicitly stated. For instance, how does the model handle uncertainties in demand and travel time? What are the implications of these assumptions on the model's applicability in real-world scenarios?
- The choice of a randomized greedy algorithm needs more justification. Why was this specific algorithm chosen over others? Comparative analysis with other potential algorithms would strengthen the argument for its use.

2. **Contributions to the Field**
    - The paper's contributions to urban planning and accessibility could be articulated more compellingly by comparing the results with other contemporary approaches. This could include a discussion on the scalability of the approach to other countries, which is currently understated. Please highlight the novel aspects of your work more clearly in relation to existing studies.
3. **Quality and Consistency of Writing, Article Flow, and Presentation of Results**
    - Some sections, especially the methodology and results sections, suffer from dense presentation. Simplifying these descriptions or using supplementary material to shift detailed algorithmic discussions could enhance readability.
    - The presentation of results (Section 4) could be enhanced by more visual representations of the changes in accessibility patterns before and after the implementation of the model.
4. **Quality and Consistency of Discussions and Conclusions**
    - The contributions could be expanded to suggest specific urban planning policies or further research areas directly stemming from the study's findings.
    - It would be beneficial if the authors discuss the limitations of their approach, particularly concerning the generalizability of the randomized greedy algorithm

across different urban settings. This could offer a more balanced view and suggest areas for further methodological enhancement.

- The discussion should more explicitly connect the findings back to the research questions and objectives. Address any unexpected results and discuss their implications.

D. Minor Comments

- There are minor typographical and grammatical errors scattered throughout the text that need correction to maintain the professionalism of the manuscript.
- Correct minor typographical errors, such as:
    - Page 3: "acounting" should be "accounting"
    - Page 3: "enahncing" should be "enhancing"
    - Page 2: "encopasses" should be "encompasses"
    - Page 3: "heteregenous" should be "heterogeneous"
    - Page 17: "deeemed" should be "deemed"
